# Salivary Adipokine and Cytokine Levels as Potential Markers for the Development of Obesity and Metabolic Disorders

**DOI:** 10.3390/ijms222111703

**Published:** 2021-10-28

**Authors:** Beata Zyśk, Lucyna Ostrowska, Joanna Smarkusz-Zarzecka

**Affiliations:** Department of Dietetics and Clinical Nutrition, Medical University of Bialystok, Ul. Mieszka I 4B, 15-054 Bialystok, Poland; lucyna.ostrowska@umb.edu.pl (L.O.); joanna.smarkusz-zarzecka@umb.edu.pl (J.S.-Z.)

**Keywords:** obesity markers, obesity diagnostics, adipokines, cytokines, saliva

## Abstract

Currently, the number of people suffering from obesity is increasing worldwide. In addition, the disease is affecting younger individuals. Therefore, it is essential to search for new diagnostic methods and markers for early assessment of the risk of obesity, metabolic disorders, and other comorbidities. The discovery of the secretory function of adipose tissue and coexistence of low-grade chronic inflammation with obesity set a new direction in this disease diagnosis using the assessment of the concentration of inflammatory markers secreted by adipose tissue. The aim of this review was to determine, based on previous findings, whether saliva can be useful in the diagnosis of obesity and its early metabolic complications and whether it can be an alternative diagnostic material to serum.

## 1. Introduction

Obesity has become a global problem of the 21st century. The World Health Organization (WHO) defines obesity as “the excessive accumulation of body fat in the body that can lead to impaired health” [1]. The latest WHO estimates showed that in 2016, more than 1.9 billion adults worldwide had excessive body weight (representing about 39% of the population), and 650 million of them were obese (13%). Between 1975 and 2016, the prevalence of obesity nearly tripled worldwide. In addition, obesity affects younger individuals. According to the WHO, in 2016, more than 350 million children and adolescents between 5 and 19 years old were found to be overweight, of which more than 124 million were obese, and in 2019 38.2 million children under 5 had excessive body weight [2].

Previous studies have shown that normal BMI values may be accompanied by an abnormal metabolic profile (metabolically obese normal weight—MONW) and, conversely, a high BMI is not always associated with the presence of metabolic disorders (metabolically healthy obese—MHO). Metabolic obesity is therefore difficult to diagnose and often recognized only when late complications occur [3,4,5]. It is of utmost importance to search for new diagnostic methods and new markers for early assessment of the risk of obesity and associated metabolic disorders so that preventive and therapeutic measures can bring the best results. A patient’s saliva analysis may prove to be a valuable innovation in the assessment of metabolic parameters due to the speed of collection, non-invasiveness, and lower cost of the test.

The aim of this review was to determine, based on previous findings, whether saliva can be useful in the diagnosis of obesity and its early metabolic complications, and to evaluate the potential markers (adipokines and/or cytokines) present in saliva.

## 2. The Role of Adipose Tissue in the Development of Obesity

The following types of adipose tissue can be distinguished in the human body: white (WAT, white adipose tissue), brown (BAT—brown adipose tissue), beige (BRITE—brown-in-white), and pink (PAT—pink adipose tissue). Each type shows a morphological and functional specificity. WAT plays an important role in the induction of inflammation and the development of metabolic disorders. Based on its anatomical location, it can be divided into visceral (VAT) and subcutaneous (SAT) adipose tissue [5,6].

The analysis of WAT deposits in terms of adipocyte growth and metabolism, angiogenesis capacity, insulin response, and adipokine and cytokine profile shows that VAT is more prone to elicit a proinflammatory response compared to SAT. VAT correlates with a higher risk of developing metabolic disorders, regardless of the BMI [4,7]. The effect of SAT on cardiometabolic risk depends on its location. Subcutaneous adipose tissue in the lower parts of the body, i.e., the gluteal subcutaneous adipose tissue (gSAT) have a protective effect and reduce these risks [4,8]. However, the results of studies on the function of abdominal subcutaneous adipose tissue (aSAT) are inconclusive [9,10,11,12]. The reason for this may be that the researchers considered only total aSAT and did not divide it into superficial and deep layers. These compartments differ in obesity at the morphological and molecular levels. Therefore, they may play a different role in the development of complications of obesity [4,13].

The development of inflammation in obesity is a result of several components, including cellular stress, excess free fatty acids and reactive oxygen species, increased lipolysis, and inadequate blood flow and hypoxia of adipocytes or their increased apoptosis, as well as increased plasma levels of lipopolysaccharide endotoxin (LPS). LPS is formed in excess in intestinal dysbiosis, which occurs in most obese patients [4,14].

During excessive energy supply, compensatory mechanisms are triggered, including the release of inflammatory mediators to stimulate adipose tissue to store more triglycerides and prevent ectopic lipid deposition. In the initial stages, it is a physiological response. However, with prolonged positive energy balance and developing obesity, the pathological proliferation of adipocytes occurs in WAT, followed by adipocyte dysfunction and activation of mechanisms leading to inflammation. Chronic low-grade inflammation develops [4]. Previous studies confirm that larger waist circumference and higher values of the BMI and WHR indices are associated with elevated serum C-reactive protein (CRP, a marker of inflammation) levels regardless of age, gender, or ethnicity [15]. A statistically significant positive relationship between BMI and salivary CRP levels was observed [16].

Not only adipocytes, but also preadipocytes, macrophages, eosinophils, mast cells, suppressor cells of myeloid origin, T lymphocytes, B lymphocytes, and fibroblasts are involved in the induction of inflammation [5]. Obesity is characterized by a significant influx of macrophages into adipose tissue. There are two extreme polarities of macrophages, i.e., M1 (classical) and M2 (alternative). Type M1 macrophages are characteristic of the adipose tissue of obese patients. This type of macrophages induces inflammation and insulin resistance by secreting the proinflammatory cytokines, including tumor necrosis factor α (TNF-α), and the interleukins IL-6, IL-12, and IL-23. In contrast, type M2 predominates in the adipose tissue of metabolically healthy, normal-weight individuals. M2-type macrophages have an opposite role to M1, namely, they protect against the development of inflammation and insulin resistance and participate in repair processes of damaged tissues. Their mechanism of action involves the secretion of IL-10, an anti-inflammatory adipokine, in significant amounts, while reducing the synthesis of pro-inflammatory interleukins, such as IL-12 and IL-23 [14,17].

Activation of M1 occurs in response to pro-inflammatory stimuli, which are the pro-inflammatory cytokines, including TNF-α and interferon γ (IFNγ), secreted by type 1 helper (Th1) lymphocytes. On the other hand, type 2 helper lymphocytes (Th2), which are characterized by high expression of anti-inflammatory interleukins i.e., IL-4, IL-13 are involved in the activation of M2 [14,17]. In addition, some adipose tissue cells (eosinophils, T-regulatory cells), adipose tissue influx cells (myeloid-derived suppressor cells—MDSCs), and other factors (peroxisome proliferator-activated receptors—PPARs) can influence macrophage polarization toward M2 and inhibit the development of inflammation and insulin resistance in adipose tissue. Other adipose tissue cells including B lymphocytes and CD8^+^ T lymphocytes influence the differentiation of macrophages into M1 type, so they promote the development of inflammation. CD4^+^ T lymphocytes can be divided into lymphocytes that promote inflammation (Th1 and Th17) and those that inhibit inflammatory responses (Th2). Mast cells, on the other hand, secrete IL-6 and IFNγ, so they have a pro-inflammatory effect [17].

Adipokines and cytokines, secreted by the WAT cells of obese patients, are mostly proinflammatory in nature. They can exhibit not only paracrine and autocrine effects, but also endocrine effects, and thus consequently affect the state and function of other tissues and the body as a whole. Proinflammatory adipokines and cytokines are a contributing factor to the development of a number of metabolic complications, including atherogenic dyslipidemia, hypertension, insulin resistance, type 2 diabetes, atherosclerosis, and non-alcoholic fatty liver disease. In contrast, adipokines with anti-inflammatory properties reduce cardiovascular risk [4,14,17].

The increased amount of adipose tissue in obese people, as well as its dysfunction, causes growth the release of free fatty acids. The increase in the concentration of free fatty acids (FFA) in the plasma contributes to the development of insulin resistance as well as the activation of the pro-inflammatory NF-κB pathway and the secretion of pro-inflammatory cytokines. The low-grade inflammation that develops in this way leads to the development of obesity-related metabolic disorders, such as non-alcoholic fatty liver disease or the formation of atherosclerotic lesions [18].

## 3. Saliva as a Potential Material in the Diagnosis of Obesity and Its Associated Disorders

The discovery of the secretory function of adipose tissue and the coexistence of low-grade chronic inflammation with obesity set a new direction in its diagnosis. It shows the possibility of evaluation of the concentration of inflammatory markers and inflammatory cytokines secreted by adipose tissue in the diagnostic process. Currently, the most common method is the determination of inflammatory markers in serum. However, blood biomarkers in saliva have been identified in obese subjects, so it is possible that saliva could be used as an alternative diagnostic material to serum. Saliva consists of water, electrolytes, different proteins, enzymes, and antibacterial substances [19]. Studies have shown a correlation between the concentration of inflammatory markers in serum and saliva. An earlier study by Tvarijonaviciute et al. showed a statistically significant strong positive correlation (r = 0.770, *p* < 0.001) between serum and salivary CRP levels [16], while another study confirmed a statistically significant moderate positive correlation (r = 0.441, *p* = 0.003) between serum and salivary resistin levels [20]. In the study of Thanakun et al., a statistically significant weak positive correlation was shown (r = 0.211, *p* = 0.018) between adiponectin levels in the given diagnostic materials but has not been shown for leptin [21]. Biomarkers enter saliva through passive diffusion, synthesis, and secretion through the salivary gland or ultrafiltration from the blood. As a result, the changes in their salivary concentration in relation to the serum may be delayed. Therefore, sometimes the biomarkers in saliva are not significantly correlated with those in serum [19].

In recent years, the relationship between salivary concentrations of selected adipokines and cytokines and obesity has been analyzed. The conducted research has shown that assessment of the salivary concentration of the following adipokines and cytokines may be considered for use in the diagnosis of obesity: adiponectin, leptin, resistin, waspin, interleukin 1β (IL-1β), IL-6, IL-8, IL-10, TNF-α, monocyte chemotactic factor, CD40/CD40L, as well as other inflammatory markers such as matrix metalloproteinase 2 (MMP-2), plasminogen activator inhibitor (PAI-1), soluble intercellular adhesion molecule 1 (sICAM-1), and pentraxin 3 (PTX3).

Adiponectin is a multimeric protein with a molecular size of 30 kDa that is mainly secreted by adipocytes of WAT. When properly secreted, adiponectin increases insulin sensitivity and protects against the development of insulin resistance and type 2 diabetes. Its beneficial effects on glucose metabolism and insulin action are associated with activation of AMP-activated kinase (AMPK) in the liver and muscle. In addition, it prevents vascular endothelial apoptosis through a pathway involving AMPK and exhibits anti-atherosclerotic effects. Furthermore, it is suspected that adiponectin participates in the control of inflammatory processes by modulating macrophage function. The lower concentration of this anti-inflammatory adipokine in adipose tissue and blood in obese compared to healthy subjects is due to inhibition of its production by released proinflammatory cytokines, as well as hypoxic conditions and oxidative stress [17,22]. Therefore, in the light of recent knowledge, salivary adiponectin concentrations may be lower in obese compared to normal-weight individuals. The results of Attlee et al. showed a statistically significant weak negative correlation (r = −0.28, *p* < 0.05) between salivary adiponectin levels and BMI among adult women. A statistically significant weak negative correlation (r = −0.28, *p* < 0.05) was also observed between salivary adiponectin levels and salivary TNF-α. In contrast, a statistically significant weak positive correlation (r = 0.37, *p* < 0.001) was shown between higher BMI and higher salivary TNF-α levels. The above results indicate that in the context of chronic inflammation associated with obesity, salivary concentrations of adiponectin and TNF-α should be analyzed simultaneously [23]. However, some of the studies conducted did not show statistically significant differences between salivary adiponectin levels in normal-weight and obese individuals [24]. Thankun et al. analyzed the correlation between salivary adiponectin levels and the components of metabolic syndrome in adults. No correlations were found [21]. In the studies of Kalyani et al., a decrease in the concentration of adiponectin in saliva was observed in the group of adults with newly diagnosed type 2 diabetes as compared to healthy people without diagnosed diabetes. These differences were statistically significant. In addition, the authors of the study found a statistically significant moderate negative correlation in patients with newly diagnosed type 2 diabetes between salivary adiponectin levels and postprandial blood glucose levels (r = −0.422, *p* = 0.02). However, no correlation was found between the concentration of this adipokine in saliva and glycosylated hemoglobin (HbA1C) and high-sensitivity CRP (hs-CRP) [25]. Moreover, Goodson et al. showed statistically significant decreased salivary adiponectin levels among children whose centile grids indicated the presence of obesity and who did not have elevated CRP levels (relative to normal-weight children) [26].

Another marker that may have applications in the diagnosis of obesity is waspin (serpin A12). It belongs to the serpin family, serine protease inhibitors. It was first identified in 2000 in the VAT of rats with obesity and type 2 diabetes. In humans, it is produced in the liver, adipose tissue, skeletal muscle, pancreas, and skin. Waspin has been shown to decrease food intake and contribute to the reduction of proinflammatory adipokines by inhibiting inflammatory processes, which may improve insulin sensitivity. Obese individuals had higher serum waspin levels compared to healthy ones. In contrast, weight reduction resulted in decreased serum waspin levels. The increase in waspin levels may have a compensatory function in obesity, insulin resistance, and type 2 diabetes [27]. In a study by Lehmann-Kalata et al., obese individuals without comorbidities had significantly higher salivary serpin A12 levels compared to overweight and normal-weight individuals. Salivary waspin levels monitoring may be helpful in the diagnosis of insulin resistance and other metabolic disorders. In addition, a high diagnostic power of the composite parameter [TNFR1] × [serpin A12] was observed, which can help in the diagnosis of metabolic obesity (it consists of multiplied tumor necrosis factor receptor 1 and serpin A12) [28]. The number of studies on waspin and its relation to obesity is small, hence the need for further research to evaluate the potential use of this anti-inflammatory marker for the diagnosis of obesity and its comorbidities.

IL-10 is a key anti-inflammatory mediator. The results show a correlation between the presence of obesity and comorbid metabolic disorders and reduced levels of this cytokine. Studies show that it can either promote M2-type macrophage activity in adipose tissue or act directly on adipocytes and consequently reduce their inflammatory response. However, the functions of this cytokine in the context of adipose tissue regulation and its therapeutic value in obesity require further research [29]. Attlee et al. observed a weak negative, non-statistically significant correlation (r = −0.20, *p* > 0.05) between salivary IL-10 levels and waist circumference and very weak negative, non-statistically significant correlation (r = −0.17, *p* > 0.05) between salivary levels of this cytokine and BMI in the adult population [23]. Moreover, other studies have shown a reduction (not statistically significant) in the mean salivary levels of this cytokine in patients diagnosed with metabolic syndrome compared to controls. There was also a statistically significant weak negative correlation (r = −0.331, *p* < 0.05) between salivary IL-10 levels and serum triglyceride levels [30]. In addition, Goodson et al. have found statistically significant decreased salivary IL-10 levels among children with obesity and without elevated CRP levels compared to normal-weight children [24,26]. The results of Selvaraju et al. showed that the concentration of this cytokine was 7.6-fold lower in overweight or obese children compared to normal weight children, but these differences were not statistically significant [31]. In the above studies, a trend was observed in which decreased levels of IL-10 in saliva correlate with excess body weight, increased waist circumference, elevated serum triglycerides, and the occurrence of metabolic syndrome. In contrast, due to the fact that the relationships demonstrated in the above studies are not statistically significant, the decreased levels of IL-10 in saliva cannot be unequivocally defined as a marker of metabolic obesity. The data results indicate the need for further research in this direction.

The CD40/CD40L complex is a cytokine that is involved in inflammatory processes in some autoimmune diseases, such as rheumatoid arthritis and systemic lupus. In addition, it has been shown that this marker may have applications in the diagnosis of Sjogren’s syndrome. In contrast, Lehmann et al. conducted a study looking at the relationship between salivary levels of this cytokine and obesity. They observed that lower sCD40L concentrations were associated with a higher risk of developing obesity and established a cut-off point (a potential prognostic marker for this disease could be a salivary sCD40L concentration < 3.28 pg/mL) [32]. However, further research in this direction is needed.

Leptin is a peptide hormone, secreted mainly by adipose tissue cells, which is involved in weight control by regulating food intake and energy expenditure. Leptin exhibits the ability to move across the blood-brain barrier, thereby exerting anorexigenic effects on the hypothalamic centers of satiety and hunger via the arcuate nucleus. High concentrations of leptin trigger a feeling of satiety (anorexic effect), while low concentrations stimulate appetite (orexigenic effect). It also modulates sympathetic activity-dependent vasoconstriction and endothelial release of nitric oxide and angiotensin II-dependent vasoconstriction, thereby controlling blood pressure in healthy individuals. Obesity induces organ-specific leptin resistance. It is most likely due to impaired passage of leptin across the blood-brain barrier or from inhibition of the intracellular leptin signaling pathway. Therefore, despite the high concentration of leptin in the body, its effect on vascular function is impaired. Leptin resistance and obesity-induced hyperleptinemia may be factors in the development of atherosclerosis. However, in the clinical setting, the importance of hyperleptinemia as a mediator of cardiovascular risk appears to be low. Martin et al. conducted a study regarding the effect of high leptin levels on the increased risk of cardiovascular disease, but they did not report an association. In addition, leptin resistance can affect reproductive function and cause hypogonadism [17,33,34]. Jayachandran et al., conducting a study among normal-weight and overweight adolescent and adult women, found higher salivary leptin concentrations in overweight subjects [35]. Thankun et al. reported no correlation between salivary leptin levels and components of metabolic syndrome in adults [21]. In contrast, the results of Goodson et al. showed significantly elevated salivary leptin levels in obese children with coexisting elevated serum CRP levels compared to normal weight children without elevated CRP [26]. Pirsean et al. observed 3-fold higher leptin levels in overweight or obese children compared to normal-weight children. In addition, the authors analyzed the occurrence of correlation between salivary leptin and IL-6 concentrations. However, salivary concentrations of the inflammatory mediators in question were not correlated [19]. Not all studies to date have shown significant differences between salivary leptin levels in normal-weight and obese subjects, which requires further research [24].

Another adipokine whose determination in saliva may be helpful in the diagnosis of obesity and comorbid disorders is resistin. In humans, it is mainly secreted by macrophages. According to available scientific data, this adipokine is involved in the pathogenesis of inflammation. Excessively high levels of resistin lead to the development of insulin resistance and type 2 diabetes. It also affects vascular endothelial dysfunction, smooth muscle cell proliferation in blood vessels, arterial inflammation, and foam cell formation, and consequently also predisposes to atherosclerosis. In addition, it may be associated with hypertension and atherogenic dyslipidemia [36]. In the study by Lehmann-Kalata et al., subjects with BMI > 30 kg/m^2^ had higher salivary resistin levels compared to subjects with BMI < 30 kg/m^2^ [28]. Al-Rawi et al. observed a statistically significant correlation (*p* = 0.007) between salivary resistin levels and BMI. Salivary resistin levels were higher in obese patients (both in the diabetic and non-diabetic groups) compared to those without diabetes and with BMI < 30 kg/m^2^. In contrast, there was no statistically significant correlation (*p* = 0.051) between salivary levels of this adipokine and fasting blood glucose levels [37]. Dogusal et al. observed no significant differences between salivary resistin levels in obese and normal-weight children. In contrast, they found a statistically significant moderate positive correlation (r = 0.567, *p* ≤ 0.01) between resistin and TNF-α levels in the saliva [38]. In a study by Selvaraju et al., conducted in a pediatric population, significantly 2.4-fold higher salivary levels of this adipokine were found in overweight and obese children compared to normal-weight children [31].

One of the most important proinflammatory mediators is TNF-α, which is secreted by adipocytes and/or peripheral tissues. TNF-α induces the development of inflammation by participating in the generation of reactive oxygen species and activation of various transcriptional pathways. Elevated levels of this cytokine interfere with insulin signaling through serine phosphorylation, leading to the development of insulin resistance in adipocytes and peripheral tissues and type 2 diabetes [39]. The results of Attlee et al. indicate that there are statistically significant weak positive correlations between salivary TNF-α levels and waist circumference (r = 0.31, *p* < 0.001) and BMI (r = 0.37, *p* < 0.001) in adults [23]. In the study by Lehmann-Kalata et al., subjects with BMI > 30 kg/m^2^ had higher salivary TNF-α and TNF-α-R1 (TNF-α receptor 1) levels compared to subjects with BMI < 30 kg/m^2^, indicating that TNF-α levels are increased in obesity [28]. The results of a subsequent study by this author et al. showed a significant difference in median TNF-α-R1 and TNF-α-R2 concentrations between subjects with BMI > 30 kg/m^2^ and controls (with BMI < 25 kg/m^2^), where TNF-α receptors concentrations were significantly higher in obese subjects [32]. Ostrowska et al. reported statistically significant strong positive correlations (r = 0.645, *p* = 0.005) between salivary TNF-α levels and BMI and statistically significant moderate positive correlations (r = 0.499, *p* = 0.041) between salivary levels of this cytokine and waist circumference. In addition, they also found statistically significant moderate positive correlations between salivary levels of this cytokine and amount of body fat [kg] (r = 0.556, *p* = 0.021) and VAT [cm^3^] (r = 0.557, *p* = 0.020) and also statistically significant strong positive correlations (r = 0.624, *p* = 0.007) between salivary TNF-α levels and VAT/SAT ratio [40]. Moreover, Chauhan et al. showed that the mean salivary TNF-α levels were significantly higher in patients with metabolic syndrome than in controls. In addition, they observed that salivary levels of this cytokine correlated significantly moderate positively with fasting plasma glucose levels (r = 0.433, *p* < 0.05), significantly moderate positively with triglyceride levels (r = 0.494, *p* < 0.01), and significantly moderate negatively with HDL cholesterol fraction levels (r= −0.490, *p* < 0.01). They also found a significant increase in TNF-α levels with an increase in the number of components of the metabolic syndrome [30]. In addition, a study by Selvaraju et al. in children showed significantly 1.5-fold higher salivary TNF-α levels in overweight and obese children compared to normal-weight children [31]. However, Doğusal et al. not have shown an association between salivary TNF-α levels and obesity [38]. Nevertheless, the strong evidence on the relationship between the concentration of TNF-a in saliva and obesity presented in the above studies and the relatively large number of these studies indicate that the concentration of TNF-a measured in saliva is a good marker of the development of obesity and its associated metabolic disorders.

IL-6 is a cytokine that is characterized by multidirectional effects that depend on various factors. The source of expression of this cytokine determines the nature of the inflammatory response. IL-6 secreted by muscle tissue in response to exercise inhibits adipose tissue macrophage (ATM) accumulation. In contrast, this cytokine secreted by adipocytes in response to the development of obesity increases macrophage accumulation in adipose tissue [41]. A study by Pirsean et al. found that salivary IL-6 levels were 4.5-fold higher in overweight or obese children compared to normal weight children. In addition, the child with the highest degree of obesity (>99th percentile) had the highest salivary IL-6 concentrations (98 ng/mL) in the entire study group [19]. Furthermore, in a study by Selvaraju et al., conducted in a pediatric population, salivary levels of this cytokine were found to be 3.4-fold higher in overweight and obese children compared to normal weight children [31]. However, Goodson et al. did not show significant differences between salivary IL-6 levels in normal weight and obese children [26].

The next interleukin that has been shown to be associated with obesity is IL-8. This cytokine is secreted predominantly by visceral adipocytes but also by macrophages. It is involved in modulating the inflammatory response. IL-8 is an interleukin involved in neutrophil chemotaxis, and its secretion is increased in obesity. Neutrophils in adipose tissue secrete chemokines and cytokines, which facilitate macrophage infiltration and may ultimately contribute to insulin resistance [42]. In a study by Ostrowska et al., salivary IL-8 levels were significantly higher among obese women compared to normal-weight women. In addition, the researchers found a statistically significant moderate positive correlation between salivary IL-8 concentration and BMI (r = 0.494, *p* = 0.044), amount of body fat [kg] (r = 0.535, *p* = 0.027) and percentage body fat [%] (r = 0.512, *p* = 0.036) [40].

IL-1β is involved in the development of insulin resistance, coexisting with obesity. This cytokine is mainly secreted by macrophages. Causes decreased insulin sensitivity of adipose tissue by inhibiting insulin signal transduction. The adverse effects of IL1β on insulin signaling in adipocytes may be related to an increase in the inflammatory response, particularly increased cytokine/chemokine production [43]. The results of Tvarijonaviciute et al. showed that salivary IL-1β levels were 2.6-fold higher in overweight/obese children compared to normal-weight children [16].

Monocyte chemotactic protein (MCP-1/CCL2) is likely involved in both the early and late phases of atherosclerosis. Moreover, MCP-1 may be a predictor of atherosclerosis-related cardiovascular events. This chemokine promotes the development of inflammatory processes at the cellular, plasma, and tissue levels and is secreted mainly by macrophages, as well as endothelial cells, but in smaller amounts also by adipocytes. MCP-1, through binding to the CCR2 receptor, initiates a number of signaling pathways that are responsible for regulating chemotactic activation and migration of target cells [44]. The results of Lehmann-Kalata et al. showed that obese adults without comorbidities had significantly higher salivary concentrations of this proinflammatory cytokine compared to overweight and normal-weight subjects [28]. Another study by this author showed such an association compared to normal-weight only subjects as well [32]. In a study in which children were the study group, significantly 4.4-fold higher salivary MCP-1 levels were found in overweight and obese children compared to normal weight children [31]. However, there are studies, conducted on a relatively large group of children, where no such correlation was found [24,26].

MMP-2 belongs to a group of proteolytic enzymes called metalloproteinases and is synthesized by various cell types, including endothelial cells, vascular smooth muscle cells, monocytes, or macrophages. It plays an important role in vascular remodeling. Therefore, it may be a potential marker for the development of atherosclerosis and cardiovascular disease [45]. In a study by Ostrowska et al., salivary MMP-2 levels were significantly higher among obese women compared to normal weight women. Moreover, statistically significant very strong positive correlations were observed between salivary MMP-2 levels and BMI (r = 0.806, *p* = 0.000) and amount of body fat [kg] (r = 0.804, *p* = 0.000). In addition, the researchers found statistically significant strong positive correlations between salivary levels of this metalloproteinase and waist circumference (r = 0.796, *p* = 0.000), percent body fat [%] (r = 0.794, *p* = 0.000) VAT [cm^3^] (r = 0.646, *p* = 0.005) and VAT/SAT ratio (r = 0.701, *p* = 0.002) [40].

According to available data, PAI-1 can influence the physiopathological mechanisms of many metabolic disorders and diseases. It is produced by adipocytes, macrophages, monocytes, platelets, hepatocytes, fibroblasts, and vascular smooth muscle cells. In obesity, elevated levels of PAI-1 are observed, which further contributes to the development of adipose tissue. In addition, it may affect the development of cerebral vascular thrombotic diseases [46]. In a study by Lehmann-Kalata et al., adults with a BMI > 30 kg/m^2^ and no comorbidities had significantly higher PAI-1 levels compared with those with BMI < 30 kg/m^2^ [28]. The authors also included other inflammatory markers in their study that may play an important role in obesity. They observed that there were higher levels of sICAM in the saliva of obese subjects compared to overweight and normal-weight subjects, which may reflect an increased risk of leukocyte (especially monocyte) adhesion to the vascular endothelium in these subjects, resulting in the formation of foam cells in atherosclerotic plaques. They also found significantly higher salivary PTX3 levels in obese subjects compared to controls (overweight and normal-weight subjects). PTX3 is a marker used to assess cardiovascular complications and the development of atherosclerosis [28]. Other studies showed a significant increase in the concentration of sICAM and PTX3 in the saliva of obese people compared to people with normal body weight [32]. Undoubtedly, these inflammatory markers require further study in terms of their potential use in the diagnosis of obesity.

In the above-cited studies, in which the study group consisted of children, 2–3 mL of saliva was collected. For adults, 5 mL of saliva was collected. In addition, in some studies, saliva was collected for a specified period of time (5 to 20 min) and the amount excreted was calculated in milliliters per minute. The saliva was collected from the morning hours to 1 p.m. In some studies, patients had to fast at the time of saliva collection, and in some studies, they had to maintain an adequate interval between food intake and collecting saliva (from 30 min to 2 h). In some studies, patients were asked to rinse their mouths before collecting saliva. During the study, the patients sat upright with the torso tilted forward. The most common type was the collection of unstimulated saliva. However, oral inflammation has not been ruled out in all studies, which may affect the levels of adipokines and cytokines in saliva.

Due to the important role of free fatty acids in the development of inflammation in obesity and its accompanying metabolic disorders, it is possible that the concentration of FFA in saliva may be a potential marker of metabolic obesity. So far, studies have been conducted in which FFA was measured in saliva, but not in the aspect of obesity [47]. Therefore, carrying out studies consisting in the determination of FFA in the saliva of obese people in the future could lead to interesting results and help in the diagnosis of obesity.

**Figure 1:**
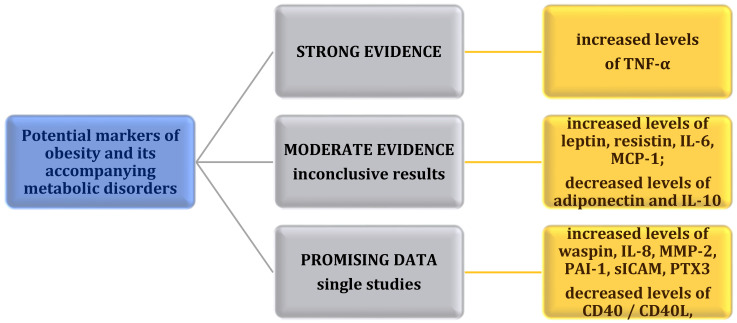
Potential markers of obesity and its accompanying metabolic disorders.

A summary of the studies cited in the review are presented in Table 1, Table 2 and Figure 1.

## 4. Conclusions

With the increasing prevalence of abnormal metabolic profiles and comorbidities of obesity in younger age groups, as in adults, it is very important to look for new diagnostic methods of obesity. The results of the cited studies, which showed a reciprocal correlation between serum concentrations of adipokines and cytokines and their salivary concentrations, demonstrate that the assessment of these markers in saliva can be not only an additional diagnostic method but also an alternative method to their assessment in serum, which can be applied to adults as well as children and adolescents. A major advantage of saliva as a diagnostic material is that it is non-invasive, which is important especially in children because of their common fear of blood sampling. However, for the results to be reliable, periodontal diseases, caries, and other oral diseases that may affect the levels of adipokines and cytokines in saliva should be ruled out.

Previous studies have identified adipokines and pro- and anti-inflammatory cytokines associated with the development of obesity as well as some metabolic disorders. The results of the presented research indicate that it may be reasonable to use the assessment of salivary concentrations of TNF-α in the diagnosis of obesity and its associated disorders and diseases. Moreover, the studies have shown that a decreased concentration of adiponectin and interleukin 10 and an increased concentration of leptin, resistin, interleukin 6, and MCP-1 could be a potential marker of obesity and its accompanying disorders. However, due to inconclusive results, more research is required in this direction. Disparate data may result from, among other things, selecting a small study group or analyzing the concentration of single markers. In addition, single studies were conducted for waspin, CD40/CD40L complex, interleukin 8, MMP-2, PAI-1, sICAM, and PTX3. However, due to the fact that results are promising, further research in this direction seems advisable. Undoubtedly, establishing reference values for specific adipokines and cytokines in saliva would enable better diagnosis of obesity and monitoring of the health status of overweight or obese individuals. In addition, further research should be pursued to evaluate the intercorrelations of adipokines and cytokines in saliva.

## Figures and Tables

**Table 1:** Summary of the studies cited in the review, which showed a correlation between the levels of selected adipokines and cytokines in saliva and the diagnostic parameters of obesity and its accompanying metabolic disorders.

Diagnostic Parameters of Obesity and Metabolic Disorders	The Levels of Adipokine/Cytokine in Saliva							
	Adiponectin	IL-10	Resistin	TNF-α	IL-8	MMP-2	Study Cited	Age Group
**↑** BMI [kg/m^2^]	↓ * **▪▪**	**↓** **▪**		↑ * **▪▪**			Attlee et al. [23]	A
			↑ * (no data)				Al-Rawi et al. [37]	A
				↑ * **▪▪▪▪**	↑ * **▪▪▪**	↑ * **▪▪▪▪▪**	Ostrowska et al. [40]	A
**↑** WC [cm]		**↓** **▪▪**		↑ * **▪▪**			Attlee et al. [23]	A
				↑ * **▪▪▪**		↑ * **▪▪▪▪**	Ostrowska et al. [40]	A
**↑** BFM [kg]				↑ * **▪▪▪**	↑ * **▪▪▪**	↑ * **▪▪▪▪▪**	Ostrowska et al. [40]	A
**↑** PBF [%]					↑ * **▪▪▪**	↑ * **▪▪▪▪**	Ostrowska et al. [40]	A
**↑** VAT [cm^3^]				↑ * **▪▪▪**		↑ * **▪▪▪▪**	Ostrowska et al. [40]	A
**↑** VAT/SAT ratio				↑ * **▪▪▪▪**		↑ * **▪▪▪▪**	Ostrowska et al. [40]	A
**↑** FPG [mg/dl]				↑ * **▪▪▪**			Chauhan et al. [30]	A
**↑** PPBS [mg/dl]	↓ * **▪▪▪**						Kalyani et al. [25]	A
**↑** TG [mg/dl]		↓ * **▪▪**		↑ * **▪▪▪**			Chauhan et al. [30]	A
**↓** HDL [mg/dl]				↓ * **▪▪▪**			Chauhan et al. [30]	A
Salivary TNF-α levels	↓ * **▪▪**						Attlee et al. [23]	A
			↑ * **▪▪▪**				Dogusal et al. [38]	Ch

Age group: A—adult, Ch—children; BMI—body mass index; WC—waist circumference; BFM—body fat mass; PBF—percent body fat; VAT—visceral adipose tissue; SAT—subcutaneous adipose tissue; FPG—fasting plasma glucose; PPBS—postprandial blood sugar; TG—triglycerides; HDLChigh-density lipoprotein cholesterol; *—statistically significant correlation (*p* < 0.05); The strength of correlation: ▪▪▪▪▪ very strong (r = 0.80–1.00); ▪▪▪▪ strong (r = 0.60–0.79); ▪▪▪ moderate (r = 0.40–0.59); ▪▪ weak (r = 0.20–0.39); ▪ very weak (r ≤ 19).

**Table 2:** Summary of the studies cited in the review, which showed a differences in the levels of adipokines/cytokines in saliva in the study and control groups.

The Levels of Adipokine/Cytokine in Saliva	Study Cited	Age Group	Differences between the Study Group and the Control Group	*p*-Value
Adipone-ctin	Kaluani et al. [25]	A	decreased adiponectin levels in the group with newly diagnosed type 2 diabetes as compared to group without diabetes;	0.04 *
	Goodson et al. [26]	Ch	decreased adiponectin levels among obese group without elevated CRP levels (relative to normal-weight group);	<0.0004 *
Waspin (serpin A12)	Lehmann-Kalata et al. [28]	A	higher waspin levels among obese group without comorbidities compared to overweight and normal weight people;	0.000004 *
IL-10	Chauhan et al. [30]	A	decreased IL-10 levels in patients diagnosed with metabolic syndrome compared to controls;	>0.05
	Goodson et al. [26]	Ch	decreased IL-10 levels among obese group without elevated CRP levels (relative to normal-weight group);	<0.0004 *
	Selvaraju et al. [31]	Ch	7.6-fold lower IL-10 concentration in overweight or obese group compared to normal weight group;	>0.05
CD40/CD40L	Lehmann et al. [32]	A	lower sCD40L concentrations in obese adults compared to normal weight subjects;	<0.001 *
Leptin	Jayachan-dran et al. [35]	A	higher leptin concentrations in overweight group;	<0.01 *
	Goodson et al. [26]	Ch	elevated leptin levels in obese group with coexisting elevated serum CRP levels compared to normal weight group without elevated CRP;	<0.0004 *
	Pirsean et al. [19]	Ch	3-fold higher leptin levels in overweight or obese group compared to normal weight group;	<0.01 *
Resistin	Al.-Rawi et al. [37]	A	higher resistin levels in obese patients (both in the diabetic and non-diabetic groups) compared to those without diabetes and obesity;	0.01 *
	Selvaraju et al. [31]	Ch	2.4-fold higher resistin levels in overweight and obese subjects compared to normal weight subjects;	<0.0001 *
	Lehmann-Kalata et al. [28]	A	higher resistin levels in subjects with BMI > 30 kg/m^2^ compared to subjects with BMI < 30 kg/m^2^;	0.013 *
TNF-α	Lehmann-Kalata et al. [28]	A	higher TNF-α levels and higher TNF-α-R1 levels in subjects with BMI > 30 kg/m^2^ compared to subjects with BMI < 30 kg/m^2^;	0.002 * 0.0003 *
	Lehmann et al. [32]	A	higher TNF-α receptors concentrations in obese subjects compared to normal weight subjects;	<0.001 * TNF-α-R1 0.02 * TNF-α-R2
	Chauhan et al. [30]	A	higher TNF-α levels in patients with metabolic syndrome than in controls; increase in TNF-α levels with an increase in the number of components of the metabolic syndrome;	<0.05 *
	Selvaraju et al. [31]	Ch	1.5-fold higher TNF-α levels in overweight and obese children compared to normal weight children;	<0.001 *
IL-6	Pirsean et al. [19]	Ch	4.5-fold higher IL-6 levels in overweight or obese children compared to normal weight children; the highest IL-6 concentrations (98 ng/mL) in the entire study group in the child with the highest degree of obesity (>99th percentile);	<0.01 *
	Selvaraju et al. [31]	Ch	3.4-fold higher IL-6 levels in overweight and obese children compared to normal weight children;	<0.001 *
IL-8	Ostrowska et al. [40]	A	higher IL-8 levels among obese women compared to normal weight women;	0.042 *
IL-1β	Tvarijona-viciute et al. [16]	Ch	2.6-fold higher IL-1β levels in overweight/obese children compared to normal weight children;	0.028 *
MCP-1/CCL2	Lehmann-Kalata et al. [28]	A	higher MCP-1 concentrations in obese adults without comorbidities compared to overweight and normal weight subjects;	0.032 *
	Lehmann et al. [32]	A	higher MCP-1 concentrations in obese adults without comorbidities compared to normal weight subjects;	<0.001 *
	Selvaraju et al. [31]	Ch	4.4-fold higher MCP-1 levels in overweight and obese subjects compared to normal weight subjects;	<0.0001 *
MMP-2	Ostrowska et al. [40]	A	higher MMP-2 levels among obese women compared to normal weight women;	0.005 *
PAI-1	Lehmann-Kalata et al. [28]	A	higher PAI-1 levels in people with a BMI > 30 kg/m^2^ and no comorbidities compared with those with BMI < 30 kg/m^2^;	0.007 *
sICAM	Lehmann-Kalata et al. [28]	A	higher sICAM levels of obese subjects compared to overweight and normal weight subjects;	0.007 *
	Lehmannet al. [32]	A	higher sICAM levels of obese subjects compared to normal weight subjects;	<0.001
PTX3	Lehmann-Kalata et al. [28]	A	higher PTX3 levels in obese subjects compared to overweight and normal weight subjects;	0.021 *
	Lehmannet al. [32]	A	higher PTX3 levels in obese subjects compared to normal weight subjects;	0.006 *

Age group: A—adult; Ch—children; *—statistically significant correlation (*p* < 0.05).

## References

[1] World Health Organization (1998). Preventing and Managing the Global Epidemic.

[2] World Health Organization (2020). Obesity and Overweight. https://www.who.int/news-room/fact-sheets/detail/obesity-and-overweight.

[3] Wang B., Zhuang R., Luo X., Yin L., Pang C., Feng T., You H., Zhai Y., Ren Y., Zhang L. (2015). Prevalence of Metabolically Healthy Obese and Metabolically Obese but Normal Weight in Adults Worldwide: A Meta-Analysis. Horm. Metab. Res..

[4] Hill H., Solt C., Foster M.T. (2018). Obesity associated disease risk: The role of inherent differences and location of adipose depots. Horm. Mol. Biol. Clin. Investig..

[5] Murawska-Ciałowicz E. (2017). Tkanka tłuszczowa-charakterystyka morfologiczna i biochemiczna różnych depozytów. Postęp. Hig. Med. Dosw..

[6] European Commission CORDIS Terapeutyczna Brunatna Tkanka Tłuszczowa w Walce z Otyłością. https://cordis.europa.eu/article/id/92120-therapeutic-brown-fat-to-tackle-obesity/pl.

[7] Bilir B.E., Güldiken S., Tunçbilek N., Demir A.M., Polat A., Bilir B. (2016). The effects of fat distribution and some adipokines on insulin resistance in subjects with prediabetes. Endokrynol. Pol..

[8] Karpe F., Pinnick K.E. (2015). Biology of upper-body and lower-body adipose tissue—Link to whole-body phenotypes. Nat. Rev. Endocrinol..

[9] Rønn P.F., Andersen G.S., Lauritzen T., Christensen D.L., Aadahl M., Carstensen B., Grarup N., Jørgensen M.E. (2020). Abdominal visceral and subcutaneous adipose tissue and associations with cardiometabolic risk in Inuit, Africans and Europeans: A cross-sectional study. BMJ Open.

[10] Matsha T.E., Ismail S., Speelman A., Hon G.M., Davids S., Erasmus R.T., Kengne A.P. (2019). Visceral and subcutaneous adipose tissue association with metabolic syndrome and its components in a South African population. Clin. Nutr. ESPEN.

[11] Lee J.J., Pedley A., Therkelsen K.E., Hoffmann U., Massaro J.M., Levy D., Long M.T. (2017). Upper Body Subcutaneous Fat Is Associated with Cardiometabolic Risk Factors. Am. J. Med..

[12] Kwon H., Kim D., Kim J.S. (2017). Body Fat Distribution and the Risk of Incident Metabolic Syndrome: A Longitudinal Cohort Study. Sci. Rep..

[13] Cancello R., Zulian A., Gentilini D., Maestrini S., Barba A.D., Invitti C., Corà D., Caselle M., Liuzzi A., Di Blasio A.M. (2013). Molecular and morphologic characterization of superficial-and deep-subcutaneous adipose tissue subdivisions in human obesity. Obesity.

[14] Neeland I.J., Ayers C.R., Rohatgi A.K., Turer A.T., Berry J.D., Das S.R., Vega G.L., Khera A., McGuire D.K., Grundy S.M. (2013). Associations of visceral and abdominal subcutaneous adipose tissue with markers of cardiac and metabolic risk in obese adults. Obesity.

[15] Choi J., Joseph L., Pilote L. (2013). Obesity and C-reactive protein in various populations: A systematic review and meta-analysis. Obes. Rev..

[16] Tvarijonaviciute A., Martinez-Lozano N., Rios R., Marcilla de Teruel M.C., Garaulet M., Cerón J.J. (2020). Saliva as a non-invasive tool for assessment of metabolic and inflammatory biomarkers in children. Clin. Nutr..

[17] Góralska M., Majewska-Szczepanik M., Szczepanik M. (2015). Mechanizmy immunologiczne towarzyszące otyłości i ich rola w zaburzeniach metabolizmu. Postep. Hig. Med. Dosw..

[18] Boden G. (2011). Obesity, Insulin Resistance and Free Fatty Acids. Curr. Opin. Endocrinol. Diabetes Obes..

[19] Pîrsean C., Neguț C., Stefan-van Staden R.I., Dinu-Pirvu C.E., Armean P., Udeanu D.I. (2019). The salivary levels of leptin and interleukin-6 as potential inflammatory markers in children obesity. PLoS ONE.

[20] Mamali I., Roupas N.D., Armeni A.K., Theodoropoulou A., Markou K.B., Georgopoulos N.A. (2012). Measurement of salivary resistin, visfatin and adiponectin levels. Peptides.

[21] Thanakun S., Watanabe H., Thaweboon S., Izumi Y. (2014). Comparison of salivary and plasma adiponectin and leptin in patients with metabolic syndrome. Diabetol. Metab. Syndr..

[22] Achari E., Jain S.K. (2017). Molecular Sciences Adiponectin, a Therapeutic Target for Obesity, Diabetes, and Endothelial Dysfunction. Int. J. Mol. Sci..

[23] Attlee A., Hasan H., AlQattan A., Sarhan N., Alshammari R., Ali S., Nabil M., Alattrash A., Raigangar V., Madkour M. (2019). Relationship of salivary adipocytokines, diet quality, physical activity, and nutrition status in adult Emirati females in United Arab Emirates. Diabetes Metab. Syndr. Clin. Res. Rev..

[24] Duffles L.F., Hermont A.P., Abreu L.G., Pordeus I.A., Silva T.A. (2019). Association between obesity and adipokines levels in saliva and gingival crevicular fluid: A systematic review and meta-analysis. J. Evid.-Based Med..

[25] Kalyani R.S., Raghunath V. (2020). Assessment of serum and salivary adiponectin levels in newly diagnosed Type II diabetes mellitus patients. J. Oral Maxillofac. Pathol..

[26] Goodson J.M., Kantarci A., Hartman M., Denis G.V., Stephens D., Hasturk H., Yaskell T., Vargas J., Wang X., Cugini M. (2014). Metabolic disease risk in children by salivary biomarker analysis. PLoS ONE.

[27] Escoté X., Gómez-Zorita S., López-Yoldi M., Milton-Laskibar I., Fernández-Quintela A., Martínez J.A., Moreno-Aliaga M.J., Portillo M.P. (2019). Role of Omentin, Vaspin, Cardiotrophin-1, TWEAK and NOV/CCN3 in Obesity and Diabetes Development. Int. J. Mol. Sci..

[28] Lehmann-Kalata A., Miechowicz I., Korybalska K., Swora-Cwynar E., Czepulis N., Łuczak J., Orzechowska Z., Grzymisławski M., Surdacka A., Witowski J. (2018). Salivary fingerprint of simple obesity. Cytokine.

[29] Saraiva M., Vieira P., O’garra A. (2020). Biology and therapeutic potential of interleukin-10. J. Exp. Med..

[30] Chauhan A., Yadav S.S., Dwivedi P., Lal N., Usman K., Khattri S. (2016). Correlation of Serum and Salivary Cytokines Level With Clinical Parameters in Metabolic Syndrome With Periodontitis. J. Clin. Lab. Anal..

[31] Selvaraju V., Babu J.R., Geetha T. (2019). Association of salivary C-reactive protein with the obesity measures and markers in children. Diabetes Metab. Syndr. Obes..

[32] Lehmann A.P., Nijakowski K., Swora-Cwynar E., Łuczak J., Czepulis N., Surdacka A. (2020). Characteristics of salivary inflammation in obesity. Polish Arch. Intern. Med..

[33] Landecho M.F., Tuero C., Valentí V., Bilbao I., De La Higuera M., Frühbeck G. (2019). Relevance of Leptin and Other Adipokines in Obesity-Associated Cardiovascular Risk. Nutrients.

[34] Martin S., Blaha M., Muse E.D., Qasim A.N., Reilly M.P., Blumenthal R.S., Nasir K., Criqui M.H., McClelland R.L., Hughes-Austin J.M. (2015). Leptin and incident cardiovascular disease: The Multi-ethnic Study of Atherosclerosis (MESA). Atherosclerosis.

[35] Jayachandran T., Srinivasan B., Padmanabhan S. (2017). Salivary leptin levels in normal weight and overweight individuals and their correlation with orthodontic tooth movement. Angle Orthod..

[36] Park H.K., Kwak M.K., Kim H.J., Ahima R.S. (2017). Linking resistin, inflammation, and cardiometabolic diseases. Korean J. Intern. Med..

[37] Al-Rawi N., Al-Marzooq F. (2017). The Relation between Periodontopathogenic Bacterial Levels and Resistin in the Saliva of Obese Type 2 Diabetic Patients. J. Diabetes Res..

[38] Doğusal G., Afacan B., Bozkurt E., Sönmez I. (2018). Gingival crevicular fluid and salivary resistin and tumor necrosis factor-alpha levels in Obese children with gingivitis. J. Periodontol..

[39] Akash M.S.H., Rehman K., Liaqat A. (2018). Tumor Necrosis Factor-Alpha: Role in Development of Insulin Resistance and Pathogenesis of Type 2 Diabetes Mellitus. J. Cell. Biochem..

[40] Ostrowska L., Gornowicz A., Pietraszewska B., Bielawski K., Bielawska A. (2020). Which salivary components can differentiate metabolic obesity?. PLoS ONE.

[41] Han M.S., White A., Perry R., Camporez J.P., Hidalgo J., Shulman G.I., Davis R.J. (2020). Regulation of adipose tissue inflammation by interleukin 6. Proc. Natl. Acad. Sci. USA.

[42] Makki K., Froguel P., Wolowczuk I. (2013). Adipose Tissue in Obesity-Related Inflammation and Insulin Resistance: Cells, Cytokines, and Chemokines. https://downloads.hindawi.com/archive/2013/139239.pdf.

[43] Bing C. (2015). Is interleukin-1b a culprit in macrophage-adipocyte crosstalk in obesity?. Adipocyte.

[44] Bianconi V., Sahebkar A., Atkin S.L., Pirro M. (2018). The regulation and importance of monocyte chemoattractant protein-1. Curr. Opin. Hematol..

[45] Miksztowicz V., Machulsky N.F., Lucero D., Fassio E., Schreier L., Berg G. (2014). Adiponectin predicts MMP-2 activity independently of obesity. Eur. J. Clin. Invest..

[46] Chen R., Yan J., Liu P., Wang Z., Wang C. (2017). Plasminogen activator inhibitor links obesity and thrombotic cerebrovascular diseases: The roles of PAI-1 and obesity on stroke. Metab. Brain Dis..

[47] Neyraud E., Cabaret S., Brignot H., Chabanet C., Labouré H., Guichard E., Berdeaux O. (2017). The basal free fatty acid concentration in human saliva is related to salivary lipolytic activity. Sci. Rep..

